# From By-Product to the Food Chain: Melon (*Cucumis melo* L.) Seeds as Potential Source for Oils

**DOI:** 10.3390/foods9101341

**Published:** 2020-09-23

**Authors:** Adrián Rabadán, M. Antónia Nunes, Silvia M. F. Bessada, José E. Pardo, M. Beatriz P. P. Oliveira, Manuel Álvarez-Ortí

**Affiliations:** 1 Higher Technical School of Agricultural and Forestry Engineering, University of Castilla-La Mancha, Campus Universitario s/n, 02071 Albacete, Spain; adrian.rabadan@uclm.es (A.R.); jose.pgonzalez@uclm.es (J.E.P.); 2 REQUIMTE/LAQV, Department of Chemical Sciences, Faculty of Pharmacy, University of Porto, R. Jorge Viterbo Ferreira, 228, 4050-313 Porto, Portugal; antonianunes.maria@gmail.com (M.A.N.); silviabessada@gmail.com (S.M.F.B.); beatoliv@ff.up.pt (M.B.P.P.O.)

**Keywords:** melon-seed oil, fruit waste, tocopherols, tocotrienols, unsaturated fatty acids, screw press

## Abstract

Fruit-processing industries annually discard large volumes of fruit by-products. Thousands of tons of melon seeds could be recovered through the year from melon production. These seeds are an excellent source of vegetable oil with significant health-promoting properties due to their unsaturated fatty acid profile and high content of specific bioactive compounds. However, little information exists about the influence of melon cultivars and oil-extraction methods on oil characteristics. In this study, oils from nine different melon cultivars were evaluated. Additionally, two oil-extraction methods (screw and hydraulic press) were studied. Results showed that melon seeds may be used as a novel source of healthy oils. Higher-quality oils were obtained with the hydraulic press; however, low yields reduced industrial interest in this method. Oils extracted from the different cultivars showed high variability in the content of linoleic (51–69%) and oleic (15–34%) acids. Regarding vitamin E, γ-tocopherol was the main isoform found in melon-seed oils (99.81–456.73 mg/kg), followed by α- and δ-tocopherols. Significant concentrations of tocotrienols (α, β, and γ) were also found. Although all cultivars showed positive attributes, principal-component analysis (PCA) showed that Honey Dew and Blanco de Ribatejo could be specifically considered as a potential source of polyunsaturated oils with high concentrations of vitamin E.

## 1. Introduction

The melon fruit (*Cucumis melo* L.) belongs to the *Cucurbitaceae* family, and it is grown in tropical and subtropical regions of the world. Global melon production has continuously risen in the last decade, reaching the current annual production of about 31.2 × 10^6^ tons. Melon processing in the industry generates large quantities of by-products that are usually discarded. Within those by-products, melon seeds account for 10% of total melon weight [1]. However, melon seeds are not considered as waste in all regions of the world. In some Arabian countries, they are roasted and directly consumed [2], and in India they are dried and used to add flavor to traditional dishes and desserts [3]. This traditional use of seeds is not applied to melon production in Europe, where melon seeds are rarely used in the food chain.

Previous studies carried out mainly on melons grown in some developing countries confirmed the interest in melon seeds as a possible functional ingredient [3,4,5]. In this regard, the nutritional composition of melon-seed cultivars grown in different countries, including Egypt [6], Brazil [7,8,9], Tunisia [10], and China [11], was studied. With regard to its nutritional content, melon seeds were found to be a rich source of proteins (14.9–27.4%), lipids (25.7–30.8%), fiber (19.0–25.3%), carbohydrates (20.8–24.8%), and ashes (3.2–4.8%) [5,8,10]. Within proteins, melon seeds contain essential amino acids such as phenylalanine, isoleucine, and leucine [7,8]. However, results show important differences in the proximate and chemical composition of seeds depending on the studied melon cultivar. This is the case of Chinese hybrid ChunLi, which shows protein percentages of up to 29.9% and small concentrations of carbohydrates (5.6%) [11].

The oil content of melon seeds and the current demand for new vegetable oils have led to an excellent opportunity for the industrial production of vegetable oil from melon seeds. The selection of an appropriate extraction method is crucial for producing high-quality oils. Most previous research studied melon-seed oils extracted using solvents [5,7,9,10,11,12]. However, the use of solvents, mainly hexane, reduces oil quality and avoid their classification as virgin oils. On the other hand, the most modern method for seed-oil extraction consists of the use of supercritical fluids [3], but the production costs of this method are high, reducing its viability for industrial production. Within this framework, cold extraction based on the use of mechanical presses for oil extraction from seeds and nuts has resulted in the production of high-quality oils at affordable prices, encouraging its use for commercial purposes [13,14,15,16].

Oils from melon seeds are mainly composed of unsaturated fatty acids, where linoleic and oleic acids are predominant. Most studies reported a content of linoleic acid ranging from 64.1% to 69.0%, and oleic acid from 13.7% to 19.4% [8,10,17]. However, these percentages may differ in some cultivars whit lower levels in linoleic acid and higher in oleic acid, like those reported by da Silva and Jorge [9], where 59.0% of linoleic acid and 26.4% of oleic acid were found in an undetermined cultivar, or those obtained by De Mello, Bora, and Narain [7], where similar percentages were found in the Daimiel cultivar (cv.). Regarding saturated fatty acids, melon-seed oil generally shows low percentages, ranging from 8.7% to 10.2% [7,8,9,10,17], although in some cultivars like ChunLi, a concentration of palmitic acid of up to 23.9% was reported [11].

Beyond the well-known beneficial effects of polyunsaturated oils for human health [18], analysis of bioactive compounds in oils is crucial. Within minor components, significant amounts of tocopherols were reported in melon-seed oils. Tocopherols and tocotrienols are part of vitamin E, a potent antioxidant that was reported to protect against cancer and bone, cardiovascular, eye, nephrological, and neurological diseases [19]. In previous studies, the average total tocopherol content in melon-seed oils was variable, with amounts between 270 and 720 mg/kg [9,10,20]. γ-tocopherol was the main tocopherol isoform described in melon oils, while δ-tocopherol was reported in the Canary melon and in cv. Maazoun, but not in other cultivars [9]. For α-tocopherol, which is the most active homologous in humans, minor concentrations (22.0–68.8 mg/kg) were reported [9,10]. With regard to tocotrienols, little information exists, as they account for roughly 1% of the total studies on vitamin E [21]. The only information about tocotrienols in melon oil was provided by Górnaś, Soliven, and Segliņa [20], who reported a total concentration of 13.7 mg/kg, with the dominance of γ-tocotrienol.

Considering the high variability of the results described in the different analyzed cultivars, further analysis of the main melon cultivars grown in Europe is needed, encouraging the return of these agroindustrial residues into the food chain. In this study, nine different *C. melo* cultivars, including three types of traditional Spanish cultivar Piel de Sapo, are evaluated. Furthermore, two pressure-extraction methods were used, and the obtained results were compared regarding their availability for the industrial extraction of high-quality oils.

## 2. Materials and Methods

### 2.1. Plant Material

Nine different melon cultivars were analyzed: Amarillo Oro Canario, Arizo, Blanco de Ribatejo, Charentais, Honeydew, Piñonet, Tendral Valenciano, Tendral Verde Tardío, and Piel de Sapo. Seeds from cv. Piel de Sapo were obtained from three different conditions: traditional cultivation, organic cultivation, and seeds from Protected Geographical Indication (PGI) Melon de la Mancha, kindly supplied by the Regulatory Board of the PGI (Tomelloso, Spain). Commercial melon seeds were obtained from local suppliers in Albacete (Spain). Seeds were cleaned and washed to remove sugars and any adhered residues. Then, seeds were dried at room temperature for several days until the seeds from all cultivars reached a moisture of less than 10%.

To evaluate the proportion of the peel with respect to the total weight of the seeds, 100 seeds were selected from each cultivar and manually peeled. The peels were weighted, and the proportion of the peel was calculated as the peel weight divided by the total seed weight.

### 2.2. Oil Extraction

Oil extraction was carried out by using a Komet Oil Press CA59G screw press (IBG Monforts Oekotec GmbH & Co. KG, Monchengladbach, Germany). One kilogram of unpeeled seeds was introduced directly into the press once the barrel was heated to 100 °C to ensure the correct extraction of oil [14]. Medium rotational-speed conditions were selected (49 rpm). Additionally, seeds from cv. Piel de Sapo PGI were subjected to extraction by using a hydraulic press (MECAMAQ model DEVF 80, Vila-Sana, Lleida, Spain). For extraction with the hydraulic press, 1 kg of ground unpeeled seeds was placed on the press, and the seeds were subjected to a pressure of 150 bar for 10 min. After pressing, oil was centrifuged to remove remaining solids. Oil samples were stored in dark glass bottles at 5 °C to avoid degradation until analysis.

### 2.3. Regulated Quality Parameters

Regulated quality parameters consist of free acidity and peroxide values. To determine free acidity, expressed as % of oleic acid, a solution of melon-seed oil dissolved in ethanol/ether (1:1) was titrated with a 0.1 mol/L potassium hydroxide ethanolic solution [22]. On the other hand, the peroxide value, expressed in milliequivalents of active oxygen per kilogram of oil (meq O_2_/kg), was measured according to European Union (EU) regulations [22]. Briefly, chloroform and acetic acid were added to an oil sample, mixed vigorously, and left to react with a solution of potassium iodide in the darkness. Then, the free iodine was titrated with a sodium thiosulfate solution [22].

### 2.4. Fatty Acid Profile 

Fatty acid profile was measured according to Santos et al. [23]. Briefly, 2 mL of n-hexane was added to 0.02 g of oil to obtain fatty acid methyl esters (FAME) by cold transmethylation with methanolic potassium hydroxide. Then, 200 µL of methanolic potassium hydroxide solution (2 N) was added and vigorously mixed. Then, the supernatant was carefully transferred to a glass vial and analyzed by gas chromatography in a Shimadzu GC-2010 Plus Gas Chromatograph (Shimadzu, Tokyo, Japan). This was performed using a CPSil 88 fused silica capillary column (50 m × 0.25 mm i.d.), 0.20 µm film thickness (Varian, Middelburg, The Netherlands), and helium was used as the carrier gas (120 kPa). The used temperature program was a first step of 5 min at 140 °C, followed by an increase of 5 °C/min from 140 to 220 °C, and then maintaining at 220 °C for 15 min. The temperature of the injector and detector was 250 and 270 °C, respectively, and the split ratio was 1:50 with an injection volume of 1 µL. Lastly, each FAME was identified by direct comparison with a standard mixture (FAME 37, Supelco, Bellefonte, PA, USA). All analyses were performed in duplicate, and results are expressed as the relative percentage of each FA on the basis of relative peak areas.

### 2.5. Vitamin E Determination

Analogously, vitamin E values were determined by HPLC analysis in oil samples according to Alves et al. [24]. Briefly, about 20 mg of oil was diluted in 1 mL of n-hexane (HPLC-grade, Merck, Darmstadt, Germany), where 20 µg/mL of tocol was added as internal standard. Then, 20 µL was injected to perform the separation on a normal-phase SupelcosilTM LC-SI column (3 µm; 75 × 3.0 mm; Supelco, Bellefonte, PA, USA). The used equipment was an HPLC system (Jasco, Tokyo, Japan) equipped with an AS-2057 automated injector, a PU-2089 pump, and an MD-2018 multiwavelength diode array detector (DAD) coupled with an FP-2020 fluorescence detector (Jasco, Japan). They were programmed for excitation at 290 nm and emission at 330 nm. Lastly, the identification of the compounds was accomplished by a comparison with commercial standards. Analyses were performed in duplicate, and results are expressed as mg/kg of oil.

### 2.6. Color Determination

Oil samples were filtered, and color was measured using a UV/Vis Jasco V-530 spectrophotometer (Jasco Analytical, Madrid, Spain). Basically, oil samples were placed in quartz cuvettes (1 cm path length) for analysis, using N-hexane as the blank reference. The obtained values were used to calculate CIELAB chromatic coordinates: L* (brightness), a* (red–green component), b* (yellow–blue component) as recommended by the Commission Internationale de l’Eclairage (CIE, Wien, Austria) [25].

### 2.7. Statistical Analysis

Data are expressed as mean ± standard deviation of the obtained results for the selected cultivars. Data were analyzed using the *t*-test and Duncan’s test. Statistical significance was defined for *p* < 0.05 (95% confidence level). Pearson’s correlations were examined. To perform principal-component analysis (PCA) for melon-seed cultivars, those variables that had previously shown significant differences were used. The Kaiser–Meyer–Olkin (KMO) test for sampling adequacy was used. All statistical analyses were carried out using the SPSS program v. 23.0 for Windows.

## 3. Results and Discussion

### 3.1. Oil-Extraction Yield

First, for a clearer idea about oil yield, it is important to consider seed parts that do not contain a significant composition in oil, which may contribute to reducing the yield value. In this sense, seed peel is mainly composed of carbohydrates, especially fiber [10]. The melon-seed peel constitutes about 30–40/100 g of seed weight. Significant differences were found between cultivars (Table 1). Cv. Piel de Sapo grown under conventional production (not organic) showed the lowest proportion of seed peel (30.65/100 g) followed by the organic Piel de Sapo. Cv. Piel de Sapo grown under PGI conditions showed one of the highest proportions of seed peel (39.00/100 g).

Melon seeds were identified as a good source of oil, with percentages of lipids about 30.7%–32.3% [8,9,10]. Oil yields from melon seeds were high enough to encourage their use for oil-production purposes when a screw press is used (Table 1). Obtained yields using the screw press were statistically different depending on the considered cultivar. Cvs. Piñonet and Piel de Sapo PGI showed the highest values, 29.90 and 26.23 g per 100 g, respectively. The lowest values were reported in the Arizo cultivar (16.95/100 g seeds). Negative but not statistically significant (*r* = −0.576, *p* = 0.64) correlation was found between peel proportion and oil yield obtained with the screw press.

### 3.2. Oil-Extraction Methods, Oil Quality, and Color

To evaluate the differences regarding extraction systems, seeds from cv. Piel de Sapo PGI were subjected to oil extraction with two presses, a hydraulic and a screw press. Extraction with the hydraulic press was performed under room temperature, while extraction with the screw press requires previous heating to obtain optimal performance. The data regarding oil yield, regulated quality (acidity and peroxide index) and color of the oils obtained are shown in Table 2. The pressure system selected for oil extraction had significant influence on oil quality and yield. Oil extraction in cv. Piel de Sapo PGI using the screw press resulted in an oil yield of 26.23/100 g, while extraction with the hydraulic press was almost four times smaller (6.80/100 g). This low yield makes the hydraulic press unsuitable for obtaining an economic benefit. Therefore, for analysis of the profile of fatty acids and vitamin E for the rest of the cultivars, only oils extracted with the screw press were used, since this could be the most appropriate method for obtaining commercial oils in the industry.

Regarding oil quality, Codex Alimentarius [26] does not have specific regulation for melon-oil quality standards. Results showed that oils obtained with the hydraulic press were of slightly better quality than that of oils obtained with the screw press. In all cases, the values in oils obtained using pressure systems were significantly lower than the values reported for melon-seed oils obtained using solvent extraction [7,11].

Melon oils show a light yellow color. Nevertheless, CIELAB color parameters showed differences attending to the used extraction method. Oils obtained with the screw press showed more intense yellow colors, with higher values for the b* parameter (Table 2). Oil extraction using the screw press requires high temperatures applied on the barrel to ensure proper oil extraction [27]. The processing temperature of screw press compared to room temperature used in hydraulic extraction may affect oil pigment content [28]. Previous studies on plant oils showed that lutein, which provides the yellow color to oils, is more resistant to high temperatures than other pigments are, such as chlorophylls [29]. Furthermore, some studies even reported an increase in the content of lutein after the thermal processing of food products due to the inactivation of enzymes responsible for oxidizing carotenoids [30]. Although the total content of carotenoids in melon oil was reported to be low [9], the balance of carotenoids in melon oil could be the reason for the observed change of color in melon oils depending on the extraction method.

### 3.3. Fatty Acids

As previously reported, melon-seed oils are mainly composed of linoleic (50.67%–69.22%) and oleic (15.23%–33.96%) acids. Saturated fatty acids, mainly palmitic and stearic, accounted for less than 15.62% in all studied cultivars (Table 3). Our data support previous results about the high variability of the fatty acid profile in melon-seed oils [5,7,8,10,11]. Some cultivars, such as Tendral Valenciano and Tendral Verde, showed a high content of linoleic acid, 69.22% and 69.15%, respectively, in comparison with cv. Piel de Sapo PGI, which showed the lowest values. As widely reported in other unsaturated plant oils, linoleic and oleic content were negatively correlated [31,32].

Extraction method had no effect on the fatty acid composition of the oils. When seeds from cv. Piel de Sapo PGI were used, oils extracted with the screw and hydraulic presses showed slight differences in fatty acid profile (Table 3). Similarly, small differences in the content of linoleic acid were found when the results for cv. Honey Dew were compared to those of the study of Bora, Narain, and de MeIio [5], who used solvent extraction for the same cultivar. In all cases, the differences reported from the cultivars were determinants compared to those small differences that could be attributed to the oil-extraction method.

### 3.4. Vitamin E

Regarding vitamin E content, the studied melon-seed oils showed significant differences (Table 4). Cvs. Honey Dew and Blanco de Ribatejo showed the highest contents of vitamin E, with 530.62 and 468.19 mg/kg, respectively. γ- and α-tocopherols were the main components of vitamin E. The amounts of α-tocopherol (37.42–74.71 mg/kg) was significantly higher than those in some previous studies for specific cultivars [9,10], but were in accordance with the data reported by Górnaś and Rudzińska [12]. α-tocopherol is crucial for oil quality, as it is the form preferentially absorbed and accumulated in humans. Cv. Tendral valenciano, which showed a small concentration of vitamin E, was, however, the one with the highest content in α-tocopherol. As reported with regard to oils from dicotyledonous plants, tocotrienol content was low compared to tocopherol content [33]; however, it was higher than that reported in some oils with similar fatty acid profiles, such as walnut oil [34,35]. γ-tocotrienol was the main tocotrienol found, in agreement with the study of Górnaś, Soliven, and Segliņa [20]. The total concentration of vitamin E in melon oils and the content of the specific isoforms could be used for the authentication of products containing melon oils and flours as functional ingredients.

Oil-seed extraction methods significantly affect the concentration of tocopherols in oils, especially if solvent extraction is compared to pressing [36,37,38]. Our results showed that significant differences appear in the concentration of tocopherol and tocotrienol forms, and in the total content of vitamin E. The vitamin E content of the Piel de Sapo PGI cultivar, extracted by hydraulic pressing, was lower than the content obtained with screw pressing; however, as reported for fatty acids, cultivar had a larger effect on vitamin E content than extraction method did.

### 3.5. Principal-Component Analysis

**Figure 1:**
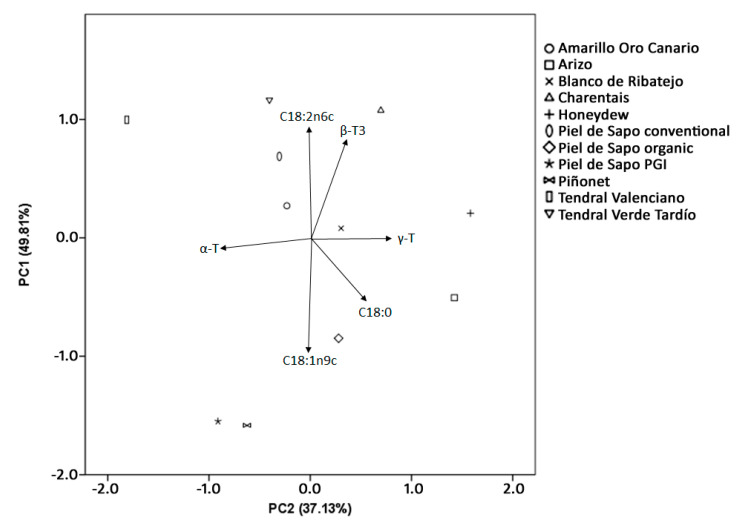
Principal-component analysis of oils from selected melon-seed cultivars.

The reported variability in the oil composition of cultivars is reflected in principal-component analysis (Figure 1). Principal Component 1 (PC1) was mainly composed of fatty acids (C18:2n6c; C18:1n9c; C18:0) and tocotrienols (β-T3), while Principal Component 2 (CP2) was composed of tocopherols (α-T; γ-T). The negative correlation between linoleic and oleic acid was clear. Similar negative correlation could be observed for the α- and γ-tocopherols. As γ-tocopherol is the main component of vitamin E in melon-seed oils, and major interest exists for oils with a high content in unsaturated fatty acids, the cultivars in the top right of Figure 1 are the most interesting for melon-seed-oil production. These are cvs. Charentais and Honey Dew. However, the provided information also encourages the production of oils with more monounsaturated fatty acids and with a higher concentration of the most active homologous α-tocopherol. In this case, the use of cvs. Piñonet and Piel de Sapo PGI, in the bottom left of Figure 1, is preferable.

## 4. Conclusions

Melon-seed oil is proposed as a highly valuable product that can be obtained from the byproducts of the agroindustrial processing of melons. This oil showed a high content of polyunsaturated fatty acids, mainly linoleic, and a high concentration of vitamin E. However, the effect of cultivar and oil-extraction method must be considered, as they have crucial influence in melon-oil characteristics. The oil-extraction method influences oil-quality parameters and oil due to the processing temperature in screw-press extraction. However, low yields obtained by hydraulic-press extraction could be inconvenient for industries to obtain an economic benefit. On the other hand, the use of different cultivars results in oils with different degrees of unsaturation and vitamin E content. Regarding vitamin E content, melon-seed oil may be considered as a rich source of tocopherols and tocotrienols. The industrial extraction of oil from melon seeds is a feasible option to obtain high-quality oil in order to meet the current demand of vegetable oils for human nutrition.

## Figures and Tables

**Table 1:** Proportion of seed peels and oil-extraction yields obtained with screw press in selected melon cultivars. PGI, Protected Geographical Indication.

Cultivar	Peel (g/100 g seeds)	Oil-Extraction Yield (g/100 g seeds)
Amarillo Oro Canario	36.02 ± 0.47 ^e^	21.43 ± 1.65 ^de^
Arizo	39.51 ± 0.33 ^ab^	16.95 ± 1.26 ^g^
Blanco de Ribatejo	37.41 ± 0.49 ^d^	19.64 ± 0.77 ^ef^
Charentais	32.79 ± 1.19 ^f^	21.43 ± 1.21 ^de^
Honeydew	38.15 ± 0.12 ^cd^	21.67 ± 0.79 ^d^
Piñonet	33.00 ± 0.21 ^f^	29.90 ± 0.54 ^a^
Tendral Valenciano	38.45 ± 0.34 ^bcd^	19.36 ± 1.72 ^f^
Tendral Verde Tardío	39.85 ± 0.23 ^a^	18.92 ± 0.84 ^f^
Piel de Sapo conventional	30.65 ± 0.10 ^g^	24.59 ± 0.73 ^bc^
Piel de Sapo organic	32.89 ± 0.20 ^f^	24.08 ± 0.81 ^c^
Piel de Sapo PGI	39.00 ± 1.36 ^abc^	26.23 ± 1.08 ^b^

Mean ± standard deviation; ^a–g^ different letters in same column represent significant differences, *p* < 0.05 between samples.

**Table 2:** Oil yield, parameters of regulated quality, and color of melon-seed oils (cv. Piel de Sapo PGI) according to extraction method.

Parameters	Hydraulic Press	Screw Press
Oil yield	6.80 ± 0.63 ^b^	26.23 ± 1.08 ^a^
Regulated quality	-	-
Acidity	0.30 ± 0.04 ^b^	0.41 ± 0.05 ^a^
Peroxide Index	0.00	0.00
Color	-	-
L*	91.28 ± 0.59 ^b^	92.75 ± 0.60 ^a^
a*	−4.70 ± 0.41 ^a^	−7.40 ± 0.90 ^b^
b*	20.97 ± 1.44 ^b^	29.62 ± 1.46 ^a^

Mean ± standard deviation; ^a,b^ different letters in the same line represent significant differences *p* < 0.05 between samples.

**Table 3:** Fatty acid profile (g/100 g of oil) of obtained melon-seed oils. All oil samples were extracted with screw press except the last column, which shows results of fatty acid profile of oil from cv Piel de Sapo (PGI), extracted with hydraulic press.

	Amarillo Oro Canario	Arizo	Blanco de Ribatejo	Charentais	Honey Dew	Piñonet	Tendral Valenciano	Tendral Verde Tardío	Piel de Sapo Conventinal	Piel de Sapo Organic	Piel de Sapo PGI	Piel de Sapo PGI Hydraulic
C14:0	0.04 ± 0.00 ^f^	0.05 ± 0.00 ^c^	0.06 ± 0.00 ^b^	0.04 ± 0.00 ^g^	0.05 ± 0.00 ^d^	0.06 ± 0.00 ^a^	0.04 ± 0.00 ^e^	0.05 ± 0.00 ^c^	0.06 ± 0.00 ^a^	0.04 ± 0.00 ^ef^	0.04 ± 0.00 ^h^	0.04 ± 0.00 ^h^
C15:0	0.03 ± 0.00 ^f^	0.03 ± 0.00 ^ab^	0.03 ± 0.00 ^a^	0.03 ± 0.00 ^cd^	0.02 ± 0.00 ^f^	0.03 ± 0.00 ^bc^	0.03 ± 0.00 ^bc^	0.03 ± 0.00 ^d^	0.03 ± 0.00 ^cd^	0.03 ± 0.00 ^c^	0.02 ± 0.00 ^e^	0.03 ± 0.00 ^e^
C16:0	8.59 ± 0.01 ^h^	9.39 ± 0.02 ^e^	9.43 ± 0.00 ^c^	9.74 ± 0.00 ^a^	7.19 ± 0.01 ^k^	8.22 ± 0.01 ^j^	9.40 ± 0.02 ^d^	9.72 ± 0.00 ^b^	8.65 ± 0.01 ^g^	8.25 ± 0.00 ^i^	9.10 ± 0.01 ^f^	9.10 ± 0.01 ^f^
C16:1	0.09 ± 0.00 ^e^	0.08 ± 0.00 ^i^	0.09 ± 0.00 ^h^	0.10 ± 0.00 ^d^	0.06 ± 0.00 ^k^	0.08 ± 0.00 ^h^	0.10 ± 0.00 ^b^	0.10 ± 0.00 ^c^	0.08 ± 0.00 ^h^	0.08 ± 0.00 ^j^	0.12 ± 0.00 ^a^	0.12 ± 0.00 ^a^
C17:0	0.07 ± 0.00 ^cd^	0.08 ± 0.00 ^a^	0.08 ± 0.00 ^ab^	0.07 ± 0.00 ^de^	0.06 ± 0.00 ^e^	0.07 ± 0.00 ^bcd^	0.07 ± 0.00 ^cd^	0.07 ± 0.00 ^abc^	0.07 ± 0.00 ^de^	0.08 ± 0.00 ^a^	0.08 ± 0.00 ^ab^	0.07 ± 0.00 ^abc^
C17:1	0.03 ± 0.00 ^a^	0.03 ± 0.00 ^a^	0.01 ± 0.00 ^e^	0.01 ± 0.00 ^cd^	0.01 ± 0.00 ^g^	0.01 ± 0.00 ^fg^	0.02 ± 0.00 ^b^	0.01 ± 0.00 ^c^	0.01 ± 0.00 ^cde^	0.01 ± 0.00 ^f^	0.01 ± 0.00 ^cd^	0.01 ± 0.00 ^de^
C18:0	5.15 ± 0.01 ^i^	5.66 ± 0.01 ^c^	5.47 ± 0.01 ^e^	5.29 ± 0.01 ^g^	5.65 ± 0.00 ^d^	5.67 ± 0.00 ^b^	4.57 ± 0.01 ^k^	5.01 ± 0.00 ^j^	5.21 ± 0.00 ^h^	5.86 ± 0.01 ^a^	5.35 ± 0.00 ^f^	5.36 ± 0.00 ^f^
C18:1n9c	18.66 ± 0.03 ^i^	27.85 ± 0.03 ^d^	21.06 ± 0.00 ^g^	15.60 ± 0.02 ^k^	22.05 ± 0.03 ^f^	31.65 ± 0.01 ^c^	15.98 ± 0.00 ^j^	15.23 ± 0.00 ^l^	18.88 ± 0.00 ^h^	25.83 ± 0.04 ^e^	33.96 ± 0.03 ^a^	33.78 ± 0.04 ^b^
C18:2n6c	66.83 ± 0.05 ^d^	56.02 ± 0.01 ^i^	63.11 ± 0.01 ^g^	68.44 ± 0.00 ^c^	64.31 ± 0.01 ^f^	53.59 ± 0.00 ^j^	69.22 ± 0.04 ^a^	69.15 ± 0.00 ^b^	66.37 ± 0.00 ^e^	59.13 ± 0.05 ^h^	50.69 ± 0.03 ^l^	50.87 ± 0.03 ^k^
C20:0	0.17 ± 0.00 ^h^	0.28 ± 0.00 ^a^	0.22 ± 0.00 ^e^	0.24 ± 0.00 ^c^	0.18 ± 0.00 ^g^	0.23 ± 0.00 ^c^	0.18 ± 0.00 ^g^	0.21 ± 0.00 ^f^	0.22 ± 0.00 ^e^	0.25 ± 0.00 ^b^	0.23 ± 0.00 ^d^	0.23 ± 0.00 ^d^
C18:3n3	0.16 ± 0.00 ^i^	0.26 ± 0.00 ^a^	0.22 ± 0.00 ^c^	0.23 ± 0.00 ^b^	0.17 ± 0.00 ^g^	0.14 ± 0.00 ^k^	0.19 ± 0.00 ^f^	0.21 ± 0.00 ^d^	0.20 ± 0.00 ^e^	0.20 ± 0.00 ^e^	0.16 ± 0.00 ^h^	0.15 ± 0.00 ^j^
C20:1n9	0.12 ± 0.00 ^f^	0.13 ± 0.00 ^d^	0.12 ± 0.00 ^e^	0.11 ± 0.00 ^i^	0.16 ± 0.00 ^a^	0.15 ± 0.00 ^b^	0.11 ± 0.00 ^j^	0.11 ± 0.00 ^h^	0.12 ± 0.00 ^g^	0.14 ± 0.00 ^c^	0.13 ± 0.00 ^d^	0.13 ± 0.00 ^d^
C21:0	0.00 ± 0.00 ^d^	0.01 ± 0.00 ^ab^	0.01 ± 0.00 ^a^	0.01 ± 0.00 ^a^	0.00 ± 0.00 ^d^	0.00 ± 0.00 ^c^	0.00 ± 0.00 ^e^	0.01 ± 0.00 ^ab^	0.01 ± 0.00 ^ab^	0.00 ± 0.00 ^c^	0.01 ± 0.00 ^b^	0.01 ± 0.00 ^b^
C20:2	0.01 ± 0.00 ^d^	0.01 ± 0.00 ^e^	0.01 ± 0.00 ^cd^	0.01 ± 0.00 ^bc^	0.02 ± 0.00 ^a^	0.01 ± 0.00 ^e^	0.01 ± 0.00 ^b^	0.01 ± 0.00 ^b^	0.01 ± 0.00 ^bc^	0.01 ± 0.00 ^cd^	0.01 ± 0.00 ^e^	0.01 ± 0.00 ^e^
C22:0	0.03 ± 0.00 ^i^	0.07 ± 0.00 ^a^	0.05 ± 0.00 ^d^	0.05 ± 0.00 ^d^	0.04 ± 0.00 ^h^	0.05 ± 0.00 ^e^	0.04 ± 0.00 ^g^	0.05 ± 0.00 ^f^	0.05 ± 0.00 ^d^	0.05 ± 0.00 ^c^	0.06 ± 0.00 ^b^	0.06 ± 0.00 ^b^
C24:0	0.05 ± 0.00 ^cd^	0.07 ± 0.01 ^a^	0.05 ± 0.00 ^b^	0.04 ± 0.00 ^de^	0.05 ± 0.00 ^bc^	0.04 ± 0.00 ^ef^	0.04 ± 0.00 ^g^	0.04 ± 0.00 ^g^	0.04 ± 0.00 ^fg^	0.05 ± 0.00 ^bc^	0.04 ± 0.00 ^de^	0.04 ± 0.00 ^de^
SFA	14.11 ± 0.03 ^i^	15.62 ± 0.01 ^a^	15.39 ± 0.02 ^c^	15.50 ± 0.02 ^b^	13.23 ± 0.02 ^j^	14.38 ± 0.01 ^g^	14.37 ± 0.03 ^g^	15.18 ± 0.01 ^d^	14.33 ± 0.01 ^h^	14.61 ± 0.01 ^f^	14.92 ± 0.01 ^e^	14.92 ± 0.01 ^e^
MUFA	18.89 ± 0.03 ^i^	28.09 ± 0.03 ^d^	21.28 ± 0.00 ^g^	15.82 ± 0.02 ^k^	22.27 ± 0.03 ^f^	31.88 ± 0.01 ^c^	16.21 ± 0.01 ^j^	15.45 ± 0.00 ^l^	19.09 ± 0.00 ^h^	26.04 ± 0.05 ^e^	34.22 ± 0.03 ^a^	34.04 ± 0.04 ^b^
PUFA	67.00 ± 0.05 ^d^	56.29 ± 0.02 ^i^	63.34 ± 0.01 ^g^	68.69 ± 0.01 ^c^	64.50 ± 0.01 ^f^	53.74 ± 0.01 ^j^	69.43 ± 0.04 ^a^	69.37 ± 0.01 ^b^	66.58 ± 0.01 ^e^	59.35 ± 0.05 ^h^	50.86 ± 0.03 ^l^	51.03 ± 0.03 ^k^

Mean ± standard deviation; ^a–l^ different letters in same line represent significant differences; *p* < 0.05 between samples.

**Table 4:** Tocopherol and tocotrienol contents (milligrams per kilogram) of studied cultivars. All oils samples were extracted with screw press except the last row, which shows results of tocopherols and tocotrienols of oil from cv. Piel de Sapo (PGI) extracted with hydraulic press.

Cultivar	α-T	α-T3	γ-T	β-T3	γ-T3	δ-T	Total Vit E
Amarillo Oro Canario	56.04 ± 1.81 ^e^	11.38 ± 0.42 ^c^	278.26 ± 4.63 ^d^	13.94 ± 1.12 ^de^	11.31 ± 0.62 ^d^	25.84 ± 0.88 ^b^	396.76 ± 1.45 ^d^
Arizo	37.42 ± 1.16 ^h^	-	358.45 ± 0.07 ^b^	18.90 ± 0.86 ^b^	12.34 ± 0.02 ^bc^	22.43 ± 0.08 ^c^	449.55 ± 2.00 ^c^
Blanco de Ribatejo	59.95 ± 1.50 ^d^	12.62 ± 0.57 ^ab^	341.15 ± 7.53 ^c^	17.52 ± 0.63 ^c^	13.64 ± 0.58 ^a^	23.33 ± 0.88 ^c^	468.19 ± 3.37 ^b^
Charentais	38.61 ± 1.24 ^h^	-	217.39 ± 1.44 ^e^	21.26 ± 0.34 ^a^	11.91 ± 0.65 ^cd^	27.17 ± 0.58 ^a^	316.33 ± 1.38 ^e^
Honey Dew	41.63 ± 0.51 ^g^	-	456.73 ± 1.30 ^a^	18.93 ± 0.72 ^b^	-	13.34 ± 0.11 ^h^	530.62 ± 2.43 ^a^
Piñonet	61.58 ± 0.21 ^d^	12.63 ± 0.63 ^ab^	128.28 ± 0.28 ^h^	11.53 ± 0.28 ^f^	-	17.82 ± 0.40 ^e^	231.83 ± 1.24 ^jk^
Tendral Valenciano	74.71 ± 3.05 ^a^	12.87 ± 0.34 ^a^	99.81 ± 1.43 ^i^	17.20 ± 0.23 ^c^	12.79 ± 0.16 ^b^	17.80 ± 0.78 ^e^	235.18 ± 6.00 ^j^
Tendral Verde Tardío	55.11 ± 0.43 ^e^	11.91 ± 0.18 ^bc^	143.84 ± 1.10 ^g^	20.79 ± 0.13 ^a^	12.26 ± 0.53 ^bc^	19.48 ± 0.51 ^d^	263.40 ± 1.51 ^h^
Piel de sapo conventional	59.91 ± 0.21 ^d^	-	180.49 ± 4.35 ^f^	20.37 ± 0.53 ^a^	13.04 ± 0.53 ^ab^	16.26 ± 0.29 ^f^	290.06 ± 3.85 ^g^
Piel de sapo organic	52.47 ± 0.99 ^f^	-	212.81 ± 0.52 ^e^	14.24 ± 0.04 ^d^	12.99 ± 0.07 ^ab^	14.78 ± 1.08 ^g^	307.29 ± 0.39 ^f^
Piel de sapo PGI	64.86 ± 0.30 ^c^	12.60 ± 0.27 ^ab^	128.44 ± 1.77 ^h^	13.02 ± 0.10 ^e^	13.69 ± 0.15 ^a^	9.40 ± 0.22 ^i^	241.99 ± 1.67 ^i^
Piel de sapo PGI HP	68.86 ± 0.46 ^b^	-	125.35 ± 2.03 ^h^	13.45 ± 0.02 ^de^	12.70 ± 0.48 ^bc^	9.33 ± 0.79 ^i^	229.70 ± 1.24 ^k^

Mean ± standard deviation; ^a–k^ different letters in the same column represent significant differences; *p* < 0.05 between samples.
